# Evaluation of the Reliability of Radiographic and MRI Angles in Superior Femoral Epiphysiolysis: A Comparative Study

**DOI:** 10.3390/diagnostics16081208

**Published:** 2026-04-17

**Authors:** Wassim Ben Abdennebi, Andreas Tsoupras, Eugénie Barras, Viola Sbampato, Romain Dayer, Giacomo De Marco, Oscar Vazquez, Christina Steiger, Amira Dhouib, Anne Tabard-Fougère, Dimitri Ceroni

**Affiliations:** 1 Paediatric Orthopaedics and Traumatology Unit, Geneva University Hospitals, University of Geneva, 1205 Geneva, Switzerland; 2 Unit of Paediatric Radiology, Division of Radiology, Geneva University Hospitals, University of Geneva, 1205 Geneva, Switzerland; 3 Department of Radiology, Neuchâtel Hospital Network, 2000 Neuchâtel, Switzerland

**Keywords:** slipped capital femoral epiphysis, MRI, radiography, imaging comparison, inter-rater reliability

## Abstract

**Background/Objectives**: Slipped Capital Femoral Epiphysis (SCFE) is a common, serious hip disorder in children and adolescents. Two-dimensional (2D) radiography is the gold standard for diagnosis but may not fully capture the deformity’s complexity, and it is vulnerable to positioning errors. Advances in three-dimensional (3D) imaging, such as computed tomography and magnetic resonance imaging (MRI), enable more accurate assessments. This study aimed to (1) assess the inter-rater reliability of 2D radiographic and 3D MRI measurements, and (2) evaluate the correlations and agreements between these outcomes. **Methods**: Patients were randomly selected from a cohort of patients aged under 16 years old and diagnosed with SCFE between January 2000 and December 2024. Southwick angles and posterior epiphyseal slip angles on 2D radiographs were independently measured by two orthopaedic surgeons. Posterior epiphyseal slip angles on 3D MRI were independently measured by two orthopaedic surgeons and two paediatric radiologists. Relationships between the three outcomes were evaluated using the Pearson correlation coefficient (r). Inter-rater reliability and agreements between the three outcomes were evaluated using the intraclass correlation coefficient (ICC) and the standard error measurement (SEM). **Results**: A total of 35 patients (35 hips) were recruited, with a mean age of 11.8 (1.2) years old and 19/35 (54%) females. Radiographic outcomes were moderately correlated (r < 0.75, *p* < 0.01) with MRI posterior epiphyseal slip angles. MRI posterior epiphyseal slip angles were systematically greater (16° on average) than both radiographic outcomes, regardless of whether contralateral correction was applied. The inter-rater reliability of radiographic outcomes was excellent (ICC > 0.85, SEM > 5.0°) and almost perfect (ICC > 0.95, SEM = 2.5°) for the MRI posterior epiphyseal slip angles measured by the paediatric radiologists. **Conclusions**: Findings suggest that while both diagnostic methods are reliable, radiographic measurements systematically underestimate epiphyseal slip severity by approximately 16° compared to MRI. This discrepancy could impact the accuracy of disease staging, leading to potential misclassifications. This highlights the need for a more standardised approach to evaluating SCFE, especially regarding the type of imaging used for angle measurement.

## 1. Introduction

Slipped Capital Femoral Epiphysis (SCFE) is a serious hip disorder with a prevalence ranging from 0.71 to 10.8 per 100,000 in children and adolescents [1,2,3,4]. It usually affects them between 8 and 15 years old, with a male-to-female ratio of 2:1 [1]. Bilateral SCFE has been reported in 18–63% of cases, and patients with unilateral slippage have a 10–30% risk of contralateral involvement within the first 18 months.

Diagnosis primarily relies on the patient’s history and a physical examination, and it is then confirmed using radiographic imaging. Conventional radiographs remain the clinician’s first-line diagnostic tool, and the Southwick angle is currently the gold-standard measurement for assessing the severity of an SCFE [5,6]. However, the use of two-dimensional (2D) radiographs raises concerns about their ability to fully capture the complexity of SCFE, as the deformity involves torsional, varus and posterior tilt components relative to the physis [7]. Additionally, radiographs can be affected by patient positioning issues and projection errors, particularly in lateral views, where positioning is challenging due to limited joint mobility and pain in the affected hip [8]. The consequences of measurement inaccuracies are reflected in their interpretation, often resulting in an underestimation of the displacement [9], and this could delay diagnosis of the condition [10,11].

Ensuring that measurements are reproducible, both between healthcare professionals and across patients, is of utmost importance for reliable diagnostic assessments. Recent advances in computed tomography (CT) and magnetic resonance imaging (MRI) are promising earlier and more accurate detection of SCFE. Reconstructing images in the plane of maximal displacement using advanced CT and MRI post-processing algorithms provides just that. It is thus legitimate to ask whether 2D radiographic measurements of the Southwick angle correlate with those established using MRI, and whether these measurements are reproducible and reliable [12].

This study’s main objectives were (1) to assess inter-rater reliability in the measurement of the Southwick angle and the posterior epiphyseal slip angle using radiographs and the posterior epiphyseal slip angle using MRI; and (2) to calculate the correlations and agreements between these outcomes to evaluate whether there were any misestimations of the disease stage.

## 2. Materials and Methods

### 2.1. Patient Selection

After obtaining ethics approval from the local Review Board (CCER 2024-02105), we conducted a retrospective review of the medical records of all the patients younger than 16 years old admitted to our institution for an SCFE between January 2000 and December 2024. We selected those who had been evaluated using both conventional radiography and MRI. Our institutional protocol involves an initial radiographic assessment followed by systematic MRI when there is evidence of a unilateral SCFE on X-ray images or when there is a clinical suspicion in the absence of radiographic abnormalities. MRI enables a simultaneous study of the injured side and early detection of pre-slip conditions. Patients with an SCFE were eligible for study inclusion if anthropometric data (weight, height, body mass index) were documented, preoperative hip radiographs were interpretable (anteroposterior view and frog-leg or Dunn views) and they had undergone MRI. Patients for whom 3D MRI reconstruction of the femoral head was impossible, due to motion artefacts, insufficient image quality or other technical issues, were excluded from the analysis. All methods and analyses were performed according to the relevant guidelines and regulations, and this work follows the Guidelines for Reporting Reliability and Agreement Studies.

### 2.2. Radiographic Assessment

Radiographic assessments used the patient’s first-line radiographic investigations, i.e., the anteroposterior, frog-leg lateral and Dunn views of their pelvis. Radiographic analyses and measurements were made using Weasis software (Weasis Manager 3.8.2-HUG imaging).

### 2.3. MRI Assessment

All MRI examinations were performed using a 1.5-tesla T MRI system (Avanto^®^ and Sola [BE1]^®^, Siemens Healthcare AG, Erlangen, Germany). Patients were imaged using a phased-array coil around the pelvis and the spine. The field of view included the whole pelvis to enable visualisation of both hips. Children were imaged supine, with their legs straight and their feet in a neutral position. We acquired one or two sequences for each patient participating in this study: (1) a coronal 3D STIR sequence, TR 2000 ms, TE 151 ms, TI 160 ms, acceleration factor 2, FOV 350 × 350 mm, matrix size 320 × 320, parallel acceleration factor 2, slice thickness 1.2 mm, voxel size 1.1 × 1.1; and/or (2) a coronal 3D DESS sequence, TR 17.9 ms, TE 6.57 ms, TI 160 ms, acceleration factor 2, FOV 400 × 400 mm, matrix size 512 × 512, parallel acceleration factor 2, slice thickness 0.8 mm, voxel size 0.8 × 0.8. Image acquisition in the coronal plane enabled reconstruction of the images in the axial and sagittal planes along the axis of the femoral neck. Images were then examined using OsiriX MD software (v. 3.0.2, Geneva, Switzerland).

### 2.4. Primary Outcomes

**Figure 1:**
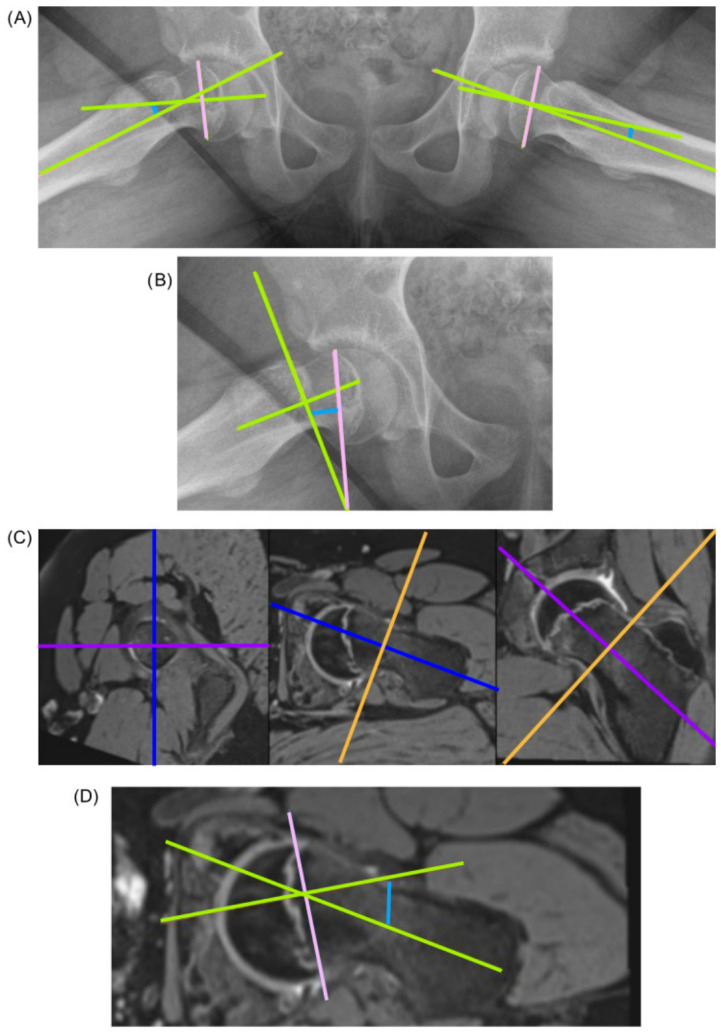
Primary outcome images. (**A**) Southwick angle evaluated using the radiographic frog-leg view; (**B**) posterior epiphyseal slip angle evaluated using the radiographic frog-leg view of the affected side; (**C**) maximum deformity plane definition using 3D MRI of the affected side; (**D**) posterior epiphyseal slip angle evaluated using MRI of the affected side.

The Southwick angle was determined by measuring the tilt of the femoral epiphysis relative to the femoral shaft on lateral radiographs. A corrective subtraction was applied, however. For unilateral SCFE, the angle of the contralateral side was subtracted; for bilateral SCFE, a 10° angle was subtracted (Figure 1A) [13]. The radiographic posterior epiphyseal slip angle was defined as the angle between the line joining the anterior and posterior corners of the epiphysis and the horizontal line passing through the femoral neck (Figure 1B). No corrective subtraction was applied here. Each radiograph measurement was independently taken and analysed by two orthopaedic surgeons.

On MRI, the femoral neck axis was determined by a line connecting three equidistant points between the lower and upper femoral neck surfaces. The epiphyseal axis was determined by the perpendicular line bisecting a point at the anterior end of the femoral epiphysis and a point at the posterior end of the femoral epiphysis (Figure 1C,D). No corrective subtraction was applied. The posterior epiphyseal slip angle was measured independently by two experienced orthopaedic surgeons and two experienced paediatric radiologists.

### 2.5. Statistical Analysis

The necessary sample size was defined using the *ICC.Sample.Size*-package in R software (v. 1.0, R Core Team, 2020), where intra-class correlation (ICC) was defined as the primary outcome. For a significance level of *p* < 0.05, a power of 90%, an anticipated ICC of 0.85 and two raters per image, the necessary sample size was calculated to be 35 patients. All analyses were performed using R software (v. 4.4.1, R Development Core Team, Vienna, Austria, 2024) and the RStudio interface (v. 2024.09.0, Posit Software, PBC). The level of significance was set at *p* < 0.05.

Inter-rater reliability was computed using the single-measure, one-way ICC, the standard error measurement (SEM) and Bland–Altman plots with levels of agreement. The presence of any proportional bias was assessed using the slope regression line fitted to the Bland–Altman plot. ICCs were interpreted as follows: ICC > 0.90 is excellent, ICC between 0.75 and 0.90 is good, ICC between 0.50 and 0.75 is moderate.

Relationships between the three measurement outcomes were evaluated using the Pearson correlation coefficient (r) reported with its associated *p*-value. Agreements between radiographic and MRI angles were evaluated using the two-way ICC, and Bland–Altman plot biases were evaluated with their respective 95% agreement limits (±1.96 standard deviations). The orthopaedic surgeons performed all the measurements and subsequent comparative analysis.

## 3. Results

### 3.1. Participants

We retrieved complete and interpretable medical data for 35 of the 111 consecutive adolescent patients hospitalised at our institution for an SCFE. This cohort comprised 19 females (54%) and had an overall mean age of 11.8 ± 1.2 years. Most patients were affected on the left side (66%), and five (14%) cases were bilateral. Seven (20%) patients were of normal weight, seven (20%) were overweight and nineteen (54%) were obese. The population is described in Table 1.

### 3.2. Inter-Rater Reliability

The inter-rater reliability of the Southwick angles and posterior epiphyseal slip angles measured from radiographs was excellent (ICC > 0.85). For the posterior epiphyseal slip angles made from MRI, inter-rater reliability was almost perfect (ICC > 0.95) between the two paediatric radiologists and excellent between the two orthopaedic surgeons (ICC = 0.91) (Table 2).

The mean absolute error (MAE) from the radiographs measured was higher for the posterior epiphyseal slip angle (6.7°) than for the Southwick angle (5.1°). This was consistent with the standard error of measurement (SEM: 6.7° [23%] vs. 5.1° [21%]) and the smallest detectable change (SDC: 13.2° [46%] vs. 9.9° [41%]).

The MAEs for MRI measurements of the posterior epiphyseal slip angle were 4.9° between the orthopaedic surgeons and just 2.7° between the paediatric radiologists. Likewise, the SEM and the SDC were also lower for the paediatric radiologists (SEM = 2.5° [6%]; SDC = 4.8° [11%], respectively) than for the orthopaedic surgeons (SEM = 6.1° [10%]; SDC = 8.2° [19%]). Notably, the relative SDC (%SDC) was lower for MRI measurements (<20%) than for radiographic ones (>40%), indicating MRI’s superior sensitivity for detecting true changes.

**Table 2:** Measurement distributions per angulation and medical speciality.

Outcomes		Mean (SD) Op1	Mean (SD) Op2	MAE (SD)	ICC [95%CI]	SEM (%)	SDC (%)
Radiographic Southwick	AS	42.8 (16.6)	40.7 (19.0)	4.6 (4.7)	0.94 [0.90; 0.96]	4.8 (13%)	9.6 (26%)
	NAS	17.0 (7.9)	13.8 (9.7)	4.3 (4.2)	0.93 [0.87; 0.96]	4.5 (26%)	8.8 (50%)
	Diff	28.2 (16.9)	28.2 (18.4)	5.2 (4.7)	0.93 [0.89; 0.95]	5.1 (21%)	9.9 (41%)
Radiographic Post. Ep. slip	AS	28.5 (16.9)	32.8 (18.9)	6.7 (6.6)	0.88 [0.77; 0.93]	6.8 (25%)	13.3 (46%)
	NAS	9.9 (7.1)	11.0 (9.5)	4.1 (4.8)	0.86 [0.78; 0.91]	4.6 (36%)	9.0 (70%)
	Diff	18.5 (12.3)	21.5 (18.5)	5.6 (4.6)	0.93 [0.86; 0.96]	5.2 (28%)	10.2 (56%)
MRI Post. Ep. slip	Ortho.	44.1 (13.8)	43.3 (13.6)	4.8 (3.6)	0.91 [0.80; 0.96]	6.1 (10%)	8.2 (19%)
	Rad.	43.5 (13.4)	45.2 (14.2)	2.7 (2.2)	0.97 [0.93; 0.99]	2.5 (6%)	4.8 (11%)

SD = standard deviation; MAE = mean absolute error; ICC = intraclass correlation coefficient; 95%CI = 95% confidence interval; SEM = standard error of measurement; SDC = smallest detectable change; AS = affected side; NAS = non-affected side; Post. Ep. = posterior epiphyseal; MRI = magnetic resonance imaging; Op1 = operator #1; Op2 = operator #2. The radiological measurements were made independently by two orthopaedic surgeons. For the MRI measurements, Ortho. = orthopaedic surgeons; Rad. = paediatric radiologists.

**Figure 2:**
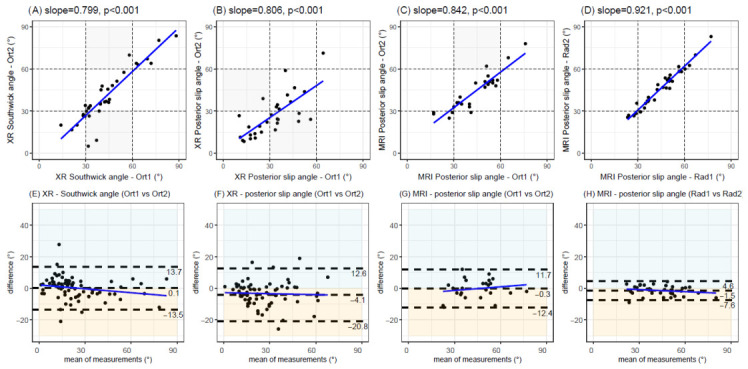
Inter-rater reliability. The top four graphs illustrate correlations between professionals (sloped lines show Pearson correlation coefficients). Grey areas highlight cases that do not match their severity category. The bottom four graphs illustrate the corresponding Bland–Altman plots with their (blue) slope regression line fitted. Dashed lines correspond to the mean differences and their 95% agreement limits (±1.96 standard deviations). Blue areas highlight cases with higher values given by professional #1; yellow areas highlight higher values given by professional #2. Ort1 = orthopaedic surgeon #1; Ort2 = orthopaedic surgeon #2; Rad1 = paediatric radiologist #1; Rad2 = paediatric radiologist #2; XR = radiography; MRI = magnetic resonance imaging; ° = degrees.

All the inter-rater measurements were highly correlated (r > 0.80, *p* < 0.001) (Figure 2A–C). The highest correlation was observed for the MRI posterior epiphyseal slip angle assessed by two paediatric radiologists (r > 0.90, *p* < 0.001) (Figure 2D).

Bland–Altman analyses revealed comparable limits of agreement for the radiographic measurements, with Southwick angles ranging from –13.5° to 13.7° (Figure 2E) and posterior epiphyseal slip angles ranging from –20.8° to 12.6° (Figure 2F).

For posterior epiphyseal slip angle measurements using MRI, inter-rater agreement was notably tighter between the paediatric radiologists (limits of agreement: –7.6° to 4.6°) (Figure 2H) than between the orthopaedic surgeons (limits of agreement: –12.4° to 11.7°) (Figure 2G).

The posterior epiphyseal slip angle measured using MRI showed excellent agreement between the orthopaedic surgeons and paediatric radiologists (ICC = 0.95, 95%CI: 0.90–0.98), with a negligible mean bias of 0.07° (from −8.2° to 8.0°) and an MAE of 3.46°.

### 3.3. Correlations and Agreements Between Measurements

**Figure 3:**
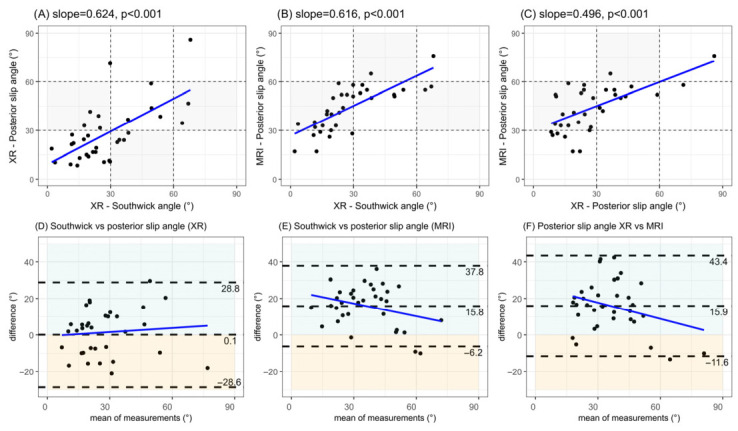
Correlations and agreements between radiographic and MRI outcomes. The top three graphs illustrate the correlations between the three primary outcomes (sloped lines show the Pearson correlation coefficients). Grey areas highlight cases that do not match their severity category. The bottom three graphs illustrate the corresponding Bland–Altman plots with their (blue) slope regression line fitted. Dashed lines correspond to the mean differences and their 95% agreement limits (±1.96 standard deviations). Blue areas highlight cases with higher values measured from MRI, yellow areas from radiography. XR = radiography; MRI = magnetic resonance imaging; ° = degrees.

Both of the angles measured radiographically were moderately correlated with the MRI posterior epiphyseal slip angle (r < 0.75, *p* < 0.01), such as the correlation between the Southwick angle and the radiographical posterior epiphyseal slip angle (Figure 3A–C).

Radiographically measured posterior epiphyseal slip angles were greater than radiographically measured Southwick angles in 15/35 (43%) hips, with a bias of −0.4° and limits of agreement ranging from −29.3° to 28.5° (Figure 3D).

Posterior epiphyseal slip angles measured using MRI were greater than radiographic Southwick angles in 32/35 (91%) hips, with a bias of 16.0° and limits of agreement ranging from –5.8° to 37.7° (Figure 3E). Similarly, posterior epiphyseal slip angles measured using MRI were greater than radiographic posterior epiphyseal slip angles in 30/35 (86%) hips, with a bias of 15.6° and limits of agreement from −11.8° to 43.0 ° (Figure 3F).

## 4. Discussion

Managing an SCFE requires an accurate assessment of physis slippage, which is crucial for treatment. Traditionally, this was done using 2D radiography, which is prone to measurement interpretation errors. The Southwick method of assessing slippage fails to accurately reflect the deformity between the epiphysis and femoral neck. Using MRI and reconstruction software creates reformatted images in the axial plane of the femoral neck, improving this measurement. Measuring the posterior epiphyseal slip angle using MRI provides a more precise 3D view of the deformity, including its posterior, varus and torsional components.

Our results showed that defining the slippage of an SCFE was more effective with MRI. In most cases, the posterior epiphyseal slip angles measured using MRI were significantly greater than both the radiographic measurements (91% were greater than Southwick angles; 86% were greater than radiographic posterior epiphyseal slip angles), with a bias of approximately 16°. These discrepancies could affect disease stage classification, leading to misestimations of severity on conventional radiographs [7]. Factors contributing to this include differences in measurement plane angles and the limited visualisation of deformities on radiographs. Additionally, maintaining the frog-lateral or Lauenstein position can be difficult or painful in severe cases [12], potentially leading to misestimations and incorrect therapeutic decisions [14]. The literature supports our finding that slips are more often underestimated [7] than overestimated when imaged incorrectly in the frog-lateral position [12]. Underestimating the slip may lead to erroneous therapeutic choices. For example, a patient with a supposedly mild slip, with an underestimation of its magnitude, local anatomy and deformity, based solely on radiographic measurements, may be treated using in situ pin fixation instead of a more aggressive procedure, such as a Southwick osteotomy or a modified Dunn procedure.

Given that both the radiographic and MRI posterior epiphyseal slip angles used in the present study are absolute measurements (not corrected taking into account the contralateral side), one would expect these two methods to show a closer agreement than the contralateral-corrected Southwick angle. However, the radiographic posterior epiphyseal slip angle still underestimated epiphyseal slip severity compared to MRI measurements, by a similar magnitude (bias 15.6°) to the Southwick angle (bias 16.0°). This suggests that the systematic underestimation of 2D radiographic measurements is not primarily due to the contralateral correction method but instead reflects its fundamental limitations in capturing a truly 3D deformity [15,16].

This study demonstrated that MRI is the most accurate and dependable method for evaluating the posterior epiphyseal slip angle in cases of an SCFE. Its inter-rater reliability score exceeding 0.95 reflected high assessment reproducibility, particularly when interpreted by specialist radiologists. In contrast, the Southwick angle and radiographic posterior epiphyseal slip showed slightly lower inter-rater reliability, likely due to difficulties associated with interpreting conventional radiographs—particularly in cases involving restricted joint mobility or significant deformities. These challenges may lead to increased measurement errors when using the Southwick method, which depends on side-to-side comparisons. Such limitations are less pronounced with assessments based on MRI. The standard error of measurement between our two paediatric radiologists using MRI was 2.5°—much smaller than the 6.7° using radiographs. Critically, the relative smallest detectable change (%SDC) was smaller using MRI (<20%) than using radiographs (>40%), highlighting MRI’s superior sensitivity for detecting true pathological changes over time. MRI’s ability to provide 3D reconstructions and enable analyses in the maximum deformity plane enhances its accuracy, especially in complex cases. Moreover, in standard clinical practice in our institution and for the purposes of this study, measurements were performed manually. To reduce inter-rater variability, an automated tool could be developed to take these measurements in the future.

Differences from MRI measurements highlighted the limitations of using radiographic measurements of the Southwick angle and the posterior epiphyseal slip angle for staging an SCFE. Radiographs, while easily available and cost effective, may lead to misinterpretations of the condition’s severity, especially in severe cases. MRI, when available, leads to more accurate assessments and avoids radiation. The variability in radiographic measurements underscores the need for standardised protocols. Although MRI is reliable and reproducible, its availability may be limited in some settings. Improving the consistency of radiographic assessments and exploring advanced imaging techniques [17], such as MRI post-processing algorithms, could enhance the diagnostic accuracy of an SCFE’s severity.

Our findings aligned with previous research reporting on how much more important the professional’s experience was [18] than the method used for measuring the posterior epiphyseal slip angle [17]. A Swedish cohort study (2015–2020) validated 91 hip radiographs against clinician-reported values and used 125 images for intra- and inter-rater measurements [17]. The mean difference between the study raters’ consensus and clinician-reported angles was 6° (±3°), with 16% of cases showing differences greater than 10°, potentially affecting treatment decisions. Intra-rater correlation was excellent, and inter-rater correlation was good [17]. The study concluded that the Southwick anteroposterior method should not be routinely used because it is less sensitive for detecting minor slips.

Other studies have evaluated the calcar femoral method [17,19], the Billing method and the head-shaft angle method as alternatives to 2D radiographic evaluation of an SFCE, and their results showed excellent inter-rater reliability for the calcar femoral and Billing methods (ICC 0.99) and high reliability for the head-shaft angle method (ICC 0.98). Intra-rater analysis revealed a mean difference of less than 1° between repeated measurements, with limits of agreement within ± 6.8°. Further studies may be needed to assess the validity of these measurements and the routine clinical use of these new techniques.

This study had some limitations. Its reliance on retrospective data could have introduced biases related to data collection and imaging quality. Excluding patients with motion artefacts or poor image quality in order to ensure accurate MRI measurements may have omitted cases that could provide insights into MRI’s limitations in SCFE assessment. Additionally, our comparison between orthopaedic surgeons’ and paediatric radiologists’ MRI measurements highlighted that raters’ professional experience matters significantly. Future research involving larger, multi-centre cohorts could provide stronger evidence on the correlations between radiographic and MRI measurements. Further studies should also explore advanced imaging technique measurements, like the Oblique Plane Deformity Angle (OPDA) or Axial Oblique Head-Neck Angle (AOHNA) [20], to assess their diagnostic capabilities.

## 5. Conclusions

The present study’s findings demonstrated that magnetic resonance imaging (MRI) is a more reliable method for assessing a Slipped Capital Femoral Epiphysis (SCFE) than traditional two-dimensional (2D) radiography. Results revealed that, in most cases, radiographic measurements of the Southwick angle and the posterior epiphyseal slip angle significantly underestimated the degree of epiphyseal slip in comparison to measurements of the posterior epiphyseal slip angle using MRI, with biases of 16.0° and 15.6°, respectively. Notably, the similar underestimation made using both radiographic methods indicates that a systematic bias is inherent to using 2D radiographic imaging rather than to the specific anatomical angle measured. Such underestimations may lead to misclassification of the condition’s severity, potentially impacting treatment decisions.

These findings raise significant concerns regarding an exclusive reliance on conventional radiographs for staging an SCFE. While radiographs remain more readily available and cost effective, their limited ability to capture a deformity’s complex three-dimensional nature can lead to inaccurate interpretations. The present study suggests that MRI provides a more comprehensive, precise assessment of the epiphyseal slip and should be performed routinely in institutions treating adolescents with an SCFE to improve treatment management and surgical planning.

## Figures and Tables

**Table 1:** Population characteristics.

Outcomes	All Patients *n* = 35 (35 Hips)
Sex, *n* (%)	F: 19 (54%); M: 16 (46%)
Age at diagnosis, year	11.8 (1.2)
Pre-pubertal, *n* (%)	17 (49%)
Pubertal, *n* (%)	17 (49%)
Post-pubertal, *n* (%)	1 (2%)
Height, cm	154.6 (14.8)
Weight, kg	60.3 (15.2)
Body mass index status	
Obese, *n* (%)	19 (54%)
Overweight, *n* (%)	7 (20%)
Normal weight, *n* (%)	7 (20%)
Underweight, *n* (%) NA	1 (3%) 1 (3%)
Side	
Left, *n* (%)	23 (66%)
Right, *n* (%)	12 (34%)
Surgery type	
Osteotomy, *n* (%)	15 (43%)
Screwing, *n* (%)	20 (57%)

To limit age- and sex-related biases on patients’ body mass indices (BMIs), these were correlated with age and computed as z-scores. Each patient’s body weight was then classified as underweight, normal, overweight or obese using the cut-offs recommended by the World Health Organization. Prepubertal patients were aged <8 years for girls, <9 for boys; pubertal patients were aged 8–13 for girls, 9–14 for boys; post-pubertal patients were ≥13 for girls, ≥14 for boys.

## Data Availability

Due to their sensitivity, the data supporting this study’s findings are not freely accessible but are available from the corresponding author upon reasonable request. The data are stored in a controlled-access repository at Geneva University Hospitals, Switzerland.

## Abbreviations

The following abbreviations are used in this manuscript:

SCFE	Slipped Capital Femoral Epiphysis
MRI	Magnetic Resonance Imaging
BMI	Body Mass Index
ICC	Intraclass Correlation Coefficient
SEM	Standard Error Measurement
